# Modelling the impact of some control strategies on the transmission dynamics of Ebola virus in human-bat population: An optimal control analysis

**DOI:** 10.1016/j.heliyon.2022.e12121

**Published:** 2022-12-07

**Authors:** Joshua Oluwasegun Agbomola, Adedapo Chris Loyinmi

**Affiliations:** a Department of Mathematics, Tulane University, New Orleans, LA, United States; b Department of Mathematics, Tai Solarin University of Education, Ijagun, Ogun State, Nigeria

**Keywords:** Ebola, Stability, Endemic, Control strategies, Optimal control

## Abstract

Qualitative research and comprehensive public awareness to nip the transmission of Ebola virus in the bud before it becomes a global threat is fast becoming imperative especially now that the Gambia Ebola virus is mutated. It is therefore necessary to consider and investigate a vector-host transmission model for possible control strategy of this deadly disease. Hence, in this study, we presented a novel and feasible human-bat (host-vector) $S_{h}E_{h}I_{h}R_{his}R_{hni}-S_{b}E_{b}I_{b}$ model which foretells the spread and severity of the Ebola virus from bats to humans to investigate the combined effects of three control strategies viz: (1) allowing specialized and designated agencies to bury deceased from Ebola infection without relatives touching or curdling the remains as usually practiced in most part of Africa as last respect for their departed love ones ($k_{1}$), (2) systematic and deliberate depopulation of bats in the metropolis (through persecution with pesticide exposure, pre capturing, chemical timber treatment for roosts destruction) to discourage hunting them for food by virtue of their proximity ($k_{2}$) and (3) immediate treatment of infected individuals in isolation ($k_{3}$). We established, among others, the endemic equilibrium, disease-free equilibrium, global and local stability, non-negativity, and boundedness of the model to prove the epidemiological feasibility of the model. The reality of the presence of optimal control remarkably influences the dynamics of transmission of the virus and simulated results also confirm the great effect of the combination of the control strategies $k_{1}$, $k_{2}$ and $k_{3}$ in flattening the curve of Ebola transmission (fig 1 – fig 8). Health workers and policy makers are better informed with fundamental precautions that could help eradicate Ebola from the populace.

## 1 Introduction

Studies have proven that the *Pteropodidae* family of fruit bats, which are the known natural host of the Ebola virus, are the source of transmission. Through close contact with a fluidic component of the body, such as discharges, blood, organs or tissues, etc., of infected animals like sick or hungry chimps, raccoons, monkeys, chimpanzees, forest antelope, or fruit bats found unwell or hunted for food in tropical regions, this virus slips into the human host. In many parts of Africa, gaming is a large-scale business, and bats which happen to be a big reservoir and transmission vector of the Ebola virus are hunted for food. Despite the concentration of earlier research on within-host control measures for the deadly infection, the threat flagged by the recent cases of Ebola infection in DR Congo after many months of WHO declaration of her Ebola-free status makes curbing of transmission of infection of the virus seems to have defied all logic. Hence, further study of possible control strategies cannot be over-emphasized as this will go a long way to curb the explosion of infection, short down possible transmission routes, remove avoidable pressure on health facilities, and subsequently save lives and costs [37].

During the year 2014–2016, Ebola virus disease (EVD) was recorded to have killed numerous people in Africa specifically in the western part. Over 16,000 analytically confirmed cases with an estimated value of 70% death rate in the affected population. The exacerbating situation of EVD was declared by the World Health Organization (WHO) on August 8, 2014, in West Africa – Public Health Emergency of International Concern (PHEIC). A sum of 28,616 cases of Ebola virus disease with 39.5% mortality rates was reported in Sierra Leone, Guinea, and Liberia in 2016. The upsurge of the Ebola virus was identified in Nigeria precisely in July 2014. The dynamic transmission of the Ebola virus disease was observed to have a breakout in seven countries are United States, Mali, the United Kingdom, Nigeria, Spain, Italy, and Senegal [33]. Eventually, secondary infection happened in Italy, Nigeria, the United States, and Italy. The virus spreads via direct contact with the body of fluidic outputs (urine, breast milk, feces, semen, vomit, amniotic fluid, sweat, saliva) of Ebola patients or the deceased body of Ebola patients, contaminated objects, infected fruits bats, or nonhuman primates which includes apes and monkeys, semen from a man who recovered from Ebola virus. And through the two treatment (Inmazeb and Ebanga) which was approved by the U.S. Food and Drug Administration (FDA) and Supportive Care, the endemic and epidemic status of Ebola virus disease in the milieu are increasingly curbed [38], [49]. Implementing methods like supportive management, isolation, and quarantine of the infected individuals in the affected geographical area, the dynamic transmission was curtailed and the case fatality rate (CFR) was kept at 40% as 8 out of the 20 infected humans yielded to the virus including a considerable number of health practitioners. The eruption mainly affected two cities in Nigeria – Port Harcourt and Lagos [1].

Numerous deterministic and stochastic models have been developed by different researchers to study the dynamic transmission, effect of vaccination, and optimization of EVD [31], [35], [50]. The following models [2], [3], [4], [5], [6], [7], [8] incorporated the transmission of deceased humans during funerals and some control measures. A novel mathematical model which revealed the relationship between chemical kinetics, epidemiology and sensitivity analysis was done by [51]. [2] created a stochastic discrete-time SEIR model for epidemiological modeling that estimated parameters from time series for daily incidence and mortality for the rise in EVD in the Democratic Republic of the Congo (DRC 1995. For their inference in the investigation of the posterior distribution of the parameters, the Markov chain Monte Carlo approach was utilized. Also, [3] analyzed the 1995 eruption in the DRC using mainly the following two sets of data (onset and death data). Their numerical simulations revealed the formulated model fits the noticed onset Ebola data at 99.95% and the noticed death data at 98.6%. The complexity of their model made them develop the Markov Chain Monte Carlo algorithm for their inference instead of Bayesian. The analysis of up-to-date infectious data of the EVD outbreak in Nigeria coupled with the impact of control interventions on the affected population was developed by [4]. Their results showed that Nigeria's apt and dynamic implementation of control interventions will hastily flatten the epidemic curve of the virus. [6] developed a model which captured the population-level impact of basic non-pharmaceutical optimal control on the 2014 Ebola upsurge. To investigate the steady directional change of human diseases both within and between the countries, [6] developed the Between-Countries Disease Spread (Be-CoDiS) mathematical model, which included the deterministic spatial-temporal model. A model that included the dynamics of direct environmental transmission of the Ebola virus disease was developed in [8], and they use the nonstandard finite difference (NSFD) method that Mickens pioneered a few years earlier. Before and after vaccination, a daily risk calculation for the risk of EVD was created by [9]. Their equation evaluated the basic transmission probability of Ebola and reduced the risk due to numerous protective measures. A typical number of mathematical models of the form non-stochastic system of nonlinear differential equations which captured the spread and control of epidemiological diseases coupled with quarantined and isolation methods of curbing the epidemic curve has been designed, among others [10], [11], [12], [13], [14], [15], [16], [17], [18], [31], [35]. [19] used a mathematical model to evaluate the number of Ebola virus disease cases of the timing and focus of introducing thousands of treatment beds as one of the control measures in every district of Sierra Leone. They estimated that 56,600 (about 95%) EVD cases were prevented in Sierra Leone as a corresponding outcome of additional treatment beds. Progressively, [20] developed a SEIR model to include optimal control strategies which involve curbing infectivity factors by the vaccination of susceptible, minimizing individuals in the exposed and infected class, and educating people about the deadly disease – EVD. And [21] formulated a mathematical model which incorporates the transmission of EVD from the dead to the living (post-death transmission). Their analysis revealed that reducing the post-death transmission of Ebola virus diseases may drastically reduce the total epidemic spread and scope considerably. Also, [22], [23] proposed a community-engaged approach to modeling Ebola. Their eight-step approach captured CARE – Community, Asset, Responsiveness, and Evaluation which provided rules for growth, implementation, and evaluation of community-engaged control strategies for the prevention, eradication, and treatment of Ebola virus and all other VHFs. A holistic approach to Ebola that did not include a mathematical model was done by [24] and this study revealed knowledge of the transmission dynamics, the pathogenesis of the disease, virus, risk factors, and epidemic control. And also they state potential control strategies which may eventually curb the epidemic level of the virus whenever there is an outbreak. Also, we have the following authors worked on optimal control strategy on some infectious diseases which are as follows cancer immunology [41], Covid-19 [40], [42], Hepatitis B virus [43], Cancer therapies [44], Corruption dynamics [45], Chronic myelogenous leukemia [46], Breast cancer [47], Ebola virus [32], [48], stochastic SIS epidemic dynamics [52] and Malaria model [28], [53].

From a set of seven potential vaccinations, two operative candidate vaccines have been developed and clinically tested through several studies [25]. Replication-defective recombinant chimpanzee adenovirus type 3, which has successfully completed analytical trials and entered Phase 3 trials, and live-replicating recombinant vesicular stomatitis virus, which has been proven effective in Phase 3 analytical studies, are the two most effective EVD vaccine candidates [26], [34]. The two operative candidate vaccines are Recombinant Vesicular Stomatitis Virus (envelop viruses) and Replication-defective recombinant chimpanzee adenovirus type 3 (non-envelop viruses). While the latter are non-enveloped viruses, by implication the antigen is absent but expressive simultaneously on the highest layer, the former is now the only EVD vaccine with demonstrated analytical efficacy in a ring-vaccination analytical study [9]. Some sacrosanct works on Ebola virus and Covid-19 which is related to fractional derivatives and its application to mathematical modeling of infectious diseases have been done by [38], [39].

As a departure from studies in existing literature on Ebola virus diseases where there is concentration on within-host transmission dynamics, we present a feasible and noble ordinary differential equations based system, $S_{h}E_{h}I_{h}R_{his}R_{hni}-S_{b}E_{b}I_{b}$ host-vector model, particularly human-bat, which predicts the transmission of the virus from bats to human to study the effectiveness of some control strategies on the transmission of this deadly infection. The control strategies considered include: allowing government approved agencies to bury deceased from Ebola infection without allowing relatives to cuddle the remains as usually practiced in most parts of Africa (cultural rites) to avoid post-death transmission, deliberate depopulation of bats in the metropolis to discourage hunting by virtue of their proximity and immediate treatment of infected individuals in isolation.

## 2 Conceptual model formulation and equations

Bifurcation analysis and dynamical behavior of a saturation incidence rate Ebola virus model are shown in [32].

### 2.1 Model description

**Figure 1:**
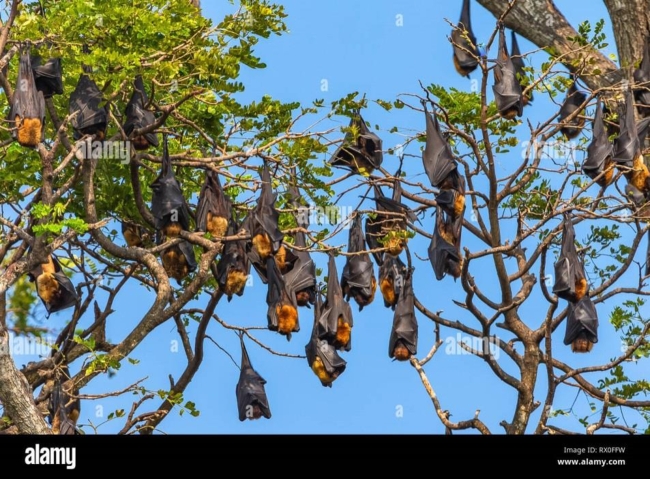
Bats on a tree.

In our model, the total population ‘*N*’ of the system has been subdivided into ‘$N_{h}$’ and ‘$N_{b}$’ for the humans and bats as indicated in equation (4). The subdivision ‘$N_{h}$’ and ‘$N_{b}$’ we proposed have the following subpopulations: the susceptible, exposed, infectious, recovered in isolation and recovered without isolation for ‘$N_{h}$’ since recovered humans either in isolation or non-isolation have the tendency of carrying the virus for some hours/days or even months. Also, we proposed susceptible, exposed and infectious for ‘$N_{b}$’ where we excluded ‘$R_{b}$’ in the bat's population with the assumption that bats cannot recover from Ebola virus (Fig. 1).

The probability distribution of those who recovered is defined as 1 as well. That is, $P(R_{hni}+R_{his})=1$ and *c* is the likelihood that a person will only fully recover in isolation once the virus has entirely left the fluidic portion of their body. The chance of a recovered human being out of isolation before the virus has completely left their body's fluidous portion is known as (1−c).

Additionally, we define the remaining variables as follows:

$\beta_{1}=$effective transmission rate between humans

$\beta_{2}=$effective transmission rate from infectious wild bats (Chiroptera) to humans

$\beta_{3}=$effective transmission rate from infectious bats to susceptible bats (Chiroptera)

*m* = saturation factor; *τ* = the treatment rate of infectious individuals.

$\phi_{1}=$the human infectious function or agent with saturation incidence

$\phi_{2}=$Bats' saturated incidence function or infection-causing agent

$\theta_{h}=$the transition rate of exposed people that become infected

$\wedge_{h}=$the recruitment/immigration rate for humans;

$\wedge_{b}=$the recruitment/immigration rate for bats

$\theta_{b}=$the transition rate of expose bats that become infectious.

$\mu_{h}=$the human's population natural death rate.

$\mu_{b}=$the natural mortality rate in bats population

*δ* = the disease-prompted mortality rate

Also, $S_{h}=$the human population that are susceptible, $E_{h}=$human population that are exposed, $I_{h}=$human population that are infectious, $R_{his}=$the human population under isolation that recovered, $R_{hni}=$the human population not under isolation that recovered, $S_{b}=$the bat's population that are susceptible, $E_{b}=$the bat's population that are exposed, $I_{b}=$the bat's population that are infective.

(1) $$\text{Where }\phi_{1}=\frac{\beta_{1}I_{h}}{1+mI_{h}}+\frac{\beta_{2}I_{b}}{1+mI_{b}}\text{ and }\phi_{2}=\frac{\beta_{3}I_{b}}{1+mI_{b}}$$

**Figure 2:**
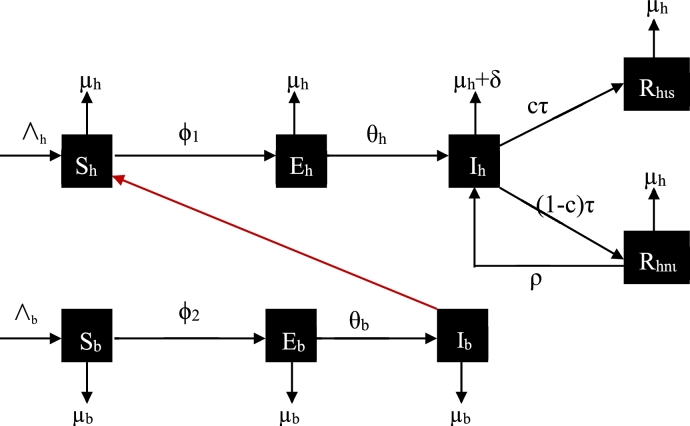
Schematic diagram of the Human-Bat.

Equation (1) are important variables. From Fig. 2, we derive the systems of equations (2) below for human population:

For human

(2) $$\begin{array}{c} \frac{dS_{h}}{dt}=\wedge_{h}-\mu_{h}S_{h}-\phi_{1}S_{h}\\ \frac{dE_{h}}{dt}=\phi_{1}S_{h}+(\mu_{h}+\theta_{h})E_{h}\\ \frac{dI_{h}}{dt}=\theta_{h}E_{h}+\rho R_{hni}-(\mu_{h}+\delta +c\tau +(1-c)\tau )I_{h}\\ \frac{dR_{his}}{dt}=c\tau I_{h}-\mu_{h}R_{his}\\ \frac{dR_{hni}}{dt}=(1-c)\tau I_{h}-(\mu_{h}+\rho )R_{hni} \end{array}\;]$$

For Bats

(3) $$\begin{array}{c} \frac{dS_{b}}{dt}=\wedge_{b}-(\mu_{b}+\phi_{2})S_{b}\\ \frac{dE_{b}}{dt}=\phi_{2}S_{b}-(\mu_{b}+\theta_{b})E_{b}\\ \frac{dI_{b}}{dt}=\theta_{b}E_{b}-\mu_{b}I_{b} \end{array}\;]$$

Where:

(4) $$\begin{array}{c} S_{h}+E_{h}+I_{h}+R_{hni}+R_{his}=N_{h},\;S_{b}+E_{b}+I_{b}=N_{b}\\ S_{h}(t=0)\geq 0,\;E_{h}(t=0)\geq 0,\;I_{h}(t=0)\geq 0,\;R_{hni}(t=0)\geq 0,\;R_{his}(t=)\geq 0\\ S_{b}(t=0)\geq 0,\;E_{b}(t=0)\geq 0,\;I_{b}(t=0)\geq 0 \end{array}\;]$$

## 3 Non-negativity and boundedness of solutions

For the epidemiological significance of the model to depict the transmission of Ebola virus, all related constraints in systems (2) and (3) must be arbitrarily non-negative. As a result, the generated analytical solutions are bounded in a feasible region and always remain positive. $\alpha_{h}=\left[\left(S_{h},E_{h},I_{h},R_{hni},R_{his}\right)\in {ℜ}_{+}^{5},N_{h}\leq \frac{\wedge_{h}}{\mu_{h}}\right]$, $\alpha_{b}=\left[\left(S_{b},E_{b},I_{b}\right)\in {ℜ}_{+}^{3},N_{b}\leq \frac{\wedge_{b}}{\mu_{b}}\right]$ and $\alpha_{T}=\left[\left(S_{h},E_{h},I_{h},R_{hni},R_{his},S_{b},E_{b},I_{b}\right)\in {ℜ}_{+}^{8},N_{h}\leq \frac{\wedge_{h}}{\mu_{h}},N_{b}\leq \frac{\wedge_{b}}{\mu_{b}}\right]$.

### 3.1 Non-negativity of solution

The solutions $S_{h}$, $E_{h}$, $I_{h}$, $R_{hni}$, $R_{his}$, $S_{b}$, $E_{b}$, $I_{b}$ of systems (2) and (3), with non-negative initial conditions, are positive for all t≥0.

Theorem 1 *The solutions* $\left(S_{h},E_{h},I_{h},R_{hni},R_{his},S_{b},I_{b},E_{b}\right)$ *of systems (2) and (3) are non - negative for all* t≻0 *, with the initial conditions as described in equation (4).*

Proof Let $\bar{\chi }=\sup\mathrm{remum}\left[t≻0:S_{h}(t)\geq 0,E_{h}(t)\geq 0,I_{h}(t)\geq 0,R_{hni}(t)\geq 0,R_{his}(t)\geq 0,S_{b}(t)\geq 0,E_{b}(t)\geq 0,I_{b}(t)\geq 0\right]$ Then, $\bar{\chi }≻0$.

Assume $S_{h}(0)\geq 0$, the first equation of (2) can be written as $\frac{dS_{h}}{dt}=\wedge_{h}-(\mu_{h}+\phi_{1})S_{h}$

$$S_{h}(t)e^{\int (\mu_{h}+\phi_{1})dt}=\int_{0}^{t}\wedge_{h}\times e^{\int (\mu_{h}+\phi_{1})dt}dt+C\;\Rightarrow \;S_{h}(t)=\wedge_{h}e^{-\int (\mu_{h}+\phi_{1})dt}\times \int_{0}^{t}e^{\int (\mu_{h}+\phi_{1})dt}dt+Ce^{-\int (\mu_{h}+\phi_{1})dt}$$

Hence $S_{h}(t)=\wedge_{h}e^{-\int (\mu_{h}+\phi_{1})dt}\times \int_{0}^{t}e^{\int (\mu_{h}+\phi_{1})dt}dt+S_{h}(0)e^{-\int (\mu_{h}+\phi_{1})dt}$.

As $S_{h}(0)\geq 0$, the sum of the positive terms $S_{h}$ is positive.

From the first equation of (3),

$$\frac{dS_{b}}{dt}=\wedge_{b}-(\mu_{b}+\phi_{2})S_{b}$$

Using a similar process above with $S_{b}(0)\geq 0$,

$$S_{b}(t)=\wedge_{b}e^{-\int (\mu_{b}+\phi_{2})dt}\times \int_{0}^{t}e^{\int (\mu_{b}+\phi_{2})dt}dt+S_{b}(0)e^{-\int (\mu_{b}+\phi_{2})dt}$$

Similarly, we can prove that the compartmental variables $E_{h}$, $I_{h}$, $R_{hni}$, $R_{his}$, $E_{b}$, $I_{b}$ are positive for all t≻0. □

### 3.2 The boundedness of solutions

Theorem 2 *All systems of equations* (2) *–* (3) *have bounded analytical solutions.*

Proof $N_{h}$ represents the total human population while $N_{b}$ represents that for bat. From equations of (4), i.e.

$$S_{h}+E_{h}+I_{h}+R_{hni}+R_{his}=N_{h}\;\text{and}\;S_{b}+E_{b}+I_{b}=N_{b}$$

$$\frac{dN_{h}}{dt}=\frac{dS_{h}}{dt}+\frac{dE_{h}}{dt}+\frac{dI_{h}}{dt}+\frac{dR_{hni}}{dt}+\frac{dR_{his}}{dt}$$

$$\frac{dN_{b}}{dt}=\frac{dS_{b}}{dt}+\frac{dE_{b}}{dt}+\frac{dI_{b}}{dt}$$

Applying the first equation in (2) and the third equation in (3), we have:

$$\frac{dN_{h}}{dt}=\wedge_{h}-\mu_{h}(S_{h}+E_{h}+I_{h}+R_{his}+R_{hni})-\delta I_{h}$$

(5) $$\frac{dN_{h}}{dt}=\wedge_{h}-\mu_{h}N_{h}\;\Rightarrow \;\frac{dN_{b}}{dt}=\wedge_{b}-\mu_{b}(S_{b}+E_{b}+I_{b})$$

(6) $$\frac{dN_{b}}{dt}=\wedge_{b}-\mu_{b}N_{b}$$

We describe that $N_{h}(t)\leq \frac{\wedge_{h}}{\mu_{h}}$ and $N_{b}\leq \frac{\wedge_{b}}{\mu_{b}}$ for all t≥0. We can then conclude from equations (5) and (6) that $\frac{dN_{h}}{dt}\leq \wedge_{h}-\mu_{h}N_{h}$, $\frac{dN_{b}}{dt}\leq \wedge_{b}-\mu_{b}N_{b}$.

Implementing Grownwall's inequality according to [7], we have:

$$N_{h}(t)\leq \frac{\wedge_{h}}{\mu_{h}}+\left(N_{h}(0)-\frac{\wedge_{h}}{\mu_{h}}\right)e^{-\mu_{h}t}\;\text{and}\;N_{b}(t)\leq \frac{\wedge_{b}}{\mu_{b}}+\left(N_{b}(0)-\frac{\wedge_{b}}{\mu_{b}}\right)e^{-\mu_{b}t}$$

And hence $N_{h}(t)\leq \frac{\wedge_{h}}{\mu_{h}}$ and $N_{b}(t)\leq \frac{\wedge_{b}}{\mu_{b}}$ for all t≥0 whenever $N_{h}(0)\leq \frac{\wedge_{h}}{\mu_{h}}$ and $N_{b}(0)\leq \frac{\wedge_{b}}{\mu_{b}}$.

This implies,

(7) $$\lim_{t\to \infty }\sup\mathrm{remum}N_{h}\leq \frac{\wedge_{h}}{\mu_{h}}\;\&\;\lim_{t\to \infty }\sup\mathrm{remum}N_{b}\leq \frac{\wedge_{b}}{\mu_{b}}$$

Equations (5) to (7) showed that $N_{h}(t)$, $N_{b}(t)$, $S_{h}(t)$, $E_{h}(t)$, $I_{h}(t)$, $R_{hni}(t)$, $R_{his}(t)$, $S_{b}(t)$, $E_{b}(t)$ and $I_{b}(t)$ are bounded, which completes the proof. □

Then

(8) $$\alpha =\left[\begin{array}{c} \left(S_{h},E_{h},I_{h},R_{hni},R_{his},S_{b},E_{b},I_{b}\right)\in {ℜ}_{+}^{8},\;N_{h}(t)\leq \frac{\wedge_{h}}{\mu_{h}},\;N_{b}(t)\leq \frac{\wedge_{b}}{\mu_{b}},\;S_{h}(t)\geq 0,\\ E_{h}(t)\geq 0,\;I_{h}(t)\geq 0,\;R_{hni}(t)\geq 0,\;R_{his}(t)\geq 0,\;S_{b}(t)\geq 0,\;E_{b}(t)\geq 0,\;I_{b}(t)\geq 0 \end{array}\right]$$

Equations (7)–(8) clearly define the region of boundedness of the model.

## 4 Basic reproductive number, $R_{0}$

The fundamental reproductive number $R_{0}$, which determines whether a disease will spread to a population or not, is a significant parameter in the epidemiological model. Here, we make all parameters more sensitive so that the value of $R_{0}$ will be less than unity. By applying the well-known generation matrix specified by [27] and respectively representing the reproductive number for humans by $R_{0H}$ and bat by $R_{0B}$, we write $R_{0h}=\rho \left(FV^{-1}\right)$.

Considering equation (2).

Let

$$\chi ={\left[E_{h},I_{h},R_{his},R_{hni}\right]}^{T},\;\chi^{⁎}=\left(F_{h}+V_{h}\right)\chi$$

We incorporated recovered humans in *χ* since they can still have Ebola virus in the fluidic part of their body for some time. So, they have the possibility of transmitting the virus to susceptible individuals. Though, the transmission dynamics is minimal in $R_{his}$ since they are in isolation.

(9) $$F=\left(\begin{array}{cccccc} 0\; & \frac{\beta_{1}\Lambda_{h}}{\mu_{h}}\; & 0\; & 0\; & 0\; & \frac{\beta_{2}\Lambda_{h}}{\mu_{h}}\\ 0\; & 0\; & 0\; & 0\; & 0\; & 0\\ 0\; & 0\; & 0\; & 0\; & 0\; & 0\\ 0\; & 0\; & 0\; & 0\; & 0\; & 0\\ 0\; & 0\; & 0\; & 0\; & 0\; & \frac{\beta_{3}\Lambda_{b}}{\mu_{b}}\\ 0\; & 0\; & 0\; & 0\; & 0\; & 0 \end{array}\right)\;\text{and}\;V=\left(\begin{array}{cccccc} (\mu_{h}+\theta_{h})\; & 0\; & 0\; & 0\; & 0\; & 0\\ -\theta_{h}\; & (\mu_{h}+\delta +c\tau +(1-c)\tau )\; & 0\; & -\rho \; & 0\; & 0\\ 0\; & -c\tau \; & \mu_{h}\; & 0\; & 0\; & 0\\ 0\; & -(1-c)\tau \; & 0\; & (\mu_{h}+\rho )\; & 0\; & 0\\ 0\; & 0\; & 0\; & 0\; & (\mu_{b}+\theta_{b})\; & 0\\ 0\; & 0\; & 0\; & 0\; & -\theta_{b}\; & \mu_{b} \end{array}\right)\;\left(\begin{array}{cccccc} \frac{\beta_{1}\Lambda_{h}\theta_{h}(\mu_{h}+\rho )}{(\mu_{h}+\theta_{h})\Omega }\; & \frac{\beta_{1}\Lambda_{h}(\mu_{h}+\rho )}{\mu_{h}\Omega }\; & 0\; & \frac{\beta_{1}\Lambda_{h}\rho }{\mu_{h}\Omega }\; & 0\; & \frac{\beta_{2}\Lambda_{b}\theta_{b}}{\mu_{b}^{2}(\mu_{b}+\theta_{b})}\\ 0\; & 0\; & 0\; & 0\; & 0\; & 0\\ 0\; & 0\; & 0\; & 0\; & 0\; & 0\\ 0\; & 0\; & 0\; & 0\; & 0\; & 0\\ 0\; & 0\; & 0\; & 0\; & \frac{\beta_{3}\Lambda_{b}\theta_{b}}{\mu_{b}^{2}(\mu_{b}+\theta_{b})}\; & \frac{\beta_{3}\Lambda_{b}}{\mu_{b}^{2}}\\ 0\; & 0\; & 0\; & 0\; & 0\; & 0 \end{array}\right)$$

where $\Omega =(\mu_{h}+\theta_{h})[(\mu_{h}+\delta +c\tau +(1-c)\tau )(\mu_{h}+\rho )-\rho (1-c)\tau ]$.

Hence, from (9) we have (10) below:

(10) $$R_{G}=\sqrt{\frac{\beta_{1}\beta_{3}\theta_{h}\theta_{b}\Lambda_{h}\Lambda_{b}(\mu_{h}+\rho )}{\mu_{b}^{2}(\mu_{b}+\theta_{b})(\mu_{h}+\theta_{h})[(\mu_{h}+\delta +c\tau +(1-c)\tau )(\mu_{h}+\rho )-\rho (1-c)\tau ]}}$$

(11) $$R_{0H}=\frac{\beta_{1}\theta_{h}\Lambda_{h}(\mu_{h}+\rho )}{\left(\mu_{h}+\theta_{h})[(\mu_{h}+\delta +c\tau +(1-c)\tau )(\mu_{h}+\rho )-\rho (1-c)\tau \right]}$$

(12) $$R_{0B}=\frac{\beta_{3}\theta_{b}\Lambda_{b}}{\mu_{b}^{2}(\mu_{b}+\theta_{b})}$$

## 5 The analysis of the Disease Free Equilibrium's (DFE) stability

The occurrence or existence of the DFE points indicates that the population as a whole is totally susceptible to the stable-state case in which the Ebola virus infection fades out.

That is $S_{h}^{0}\neq 0$ and $S_{b}^{0}\neq 0$.

At the DFE point at $E^{0}$, $S_{h}=E_{h}=I_{h}=R_{his}=R_{hni}=S_{b}=E_{b}=I_{b}=0$

$$\wedge_{h}-\mu_{h}S_{h}^{0}-\phi_{1}S_{h}^{0}=0\;\phi_{1}S_{h}^{0}-(\mu_{h}+\theta_{h})E_{h}^{0}=0\;\theta_{h}E_{h}^{0}+\rho R_{hni}^{0}-(\mu_{h}+\delta +c\tau +(1-c)\tau )I_{h}^{0}=0\;c\tau I_{h}^{0}-\mu_{h}R_{his}^{0}=0(1-c)\tau I_{h}^{0}-(\mu_{h}+\rho )R_{hni}^{0}=0\wedge_{b}-(\mu_{b}+\phi_{2})S_{b}^{0}=0\;\phi_{2}S_{b}^{0}-(\mu_{b}+\theta_{b})E_{b}=0\;\theta_{b}E_{b}^{0}-\mu_{b}I_{b}^{0}=0$$

Since $S_{h}^{0}\neq 0$ at DFE, then

(13) $$E_{h}^{0}=0,\;I_{h}^{0}=0,\;R_{his}^{0}=0,\;R_{hni}^{0}=0=E^{0}=\left(S_{h}^{0},E_{h}^{0},I_{h}^{0},R_{his}^{0},R_{hni}^{0},S_{b}^{0},E_{b}^{0},I_{b}^{0}\right)=\left(\frac{\wedge_{h}}{\mu_{b}},0,0,0,0,\frac{\wedge_{b}}{\mu_{b}},0,0\right)$$

Equation (13) defines the points at which the population is entirely susceptible.

### 5.1 Model local stability analysis

Theorem 3 *The Disease Free Equilibrium (DFE)* $E^{0}=\left(\frac{\wedge_{h}}{\mu_{h}},0,0,0,0,\frac{\wedge_{b}}{\mu_{b}},0,0\right)$ *of the system is said to be locally asymptotically stable if all eigenvalues of the systems generated Jacobian's matrix are negative real values.*

Proof The Jacobian matrix $J(S_{h},E_{h},I_{h},R_{his},R_{hni},S_{b},E_{b},I_{b})$ of systems (2) to (3) at DFE (13) is given as:

(14) $$J=\left[\begin{array}{cccccccc} -\phi_{1}-\mu_{h}\; & 0\; & \frac{-\beta_{1}S_{h}}{1+mI_{h}}\; & 0\; & 0\; & 0\; & 0\; & \frac{-\beta_{2}S_{h}}{1+mI_{b}}\\ \phi_{1}\; & -(\mu_{h}+\theta_{h})\; & \frac{\beta_{1}S_{h}}{1+mI_{h}}\; & 0\; & 0\; & 0\; & 0\; & \frac{\beta_{2}S_{h}}{1+mI_{b}}\\ 0\; & \theta_{h}\; & -(\mu_{h}+\delta +c\tau +(1-c)\tau )\; & 0\; & \rho \; & 0\; & 0\; & 0\\ 0\; & 0\; & c\tau \; & -\mu_{h}\; & 0\; & 0\; & 0\; & 0\\ 0\; & 0\; & (1-c)\tau \; & 0\; & -(\mu_{h}+\rho )\; & 0\; & 0\; & 0\\ 0\; & 0\; & 0\; & 0\; & 0\; & -(\mu_{b}+\phi_{2})\; & 0\; & \frac{-\beta_{3}S_{b}}{1+mI}\\ 0\; & 0\; & 0\; & 0\; & 0\; & \phi_{2}\; & -(\mu_{b}+\theta_{b})\; & \frac{\beta_{3}S_{h}}{1+mI_{b}}\\ 0\; & 0\; & 0\; & 0\; & 0\; & 0\; & \theta_{b}\; & -\mu_{b} \end{array}\right]$$

At DFE, $E^{0}=\left(\frac{\wedge_{h}}{\mu_{h}},0,0,0,0,\frac{\wedge_{b}}{\mu_{b}},0,0\right)$

(15) $$J=\left[\begin{array}{cccccccc} -\mu_{h}\; & 0\; & \frac{-\beta_{1}\wedge_{h}}{\mu_{h}}\; & 0\; & 0\; & 0\; & 0\; & \frac{-\beta_{2}\wedge_{h}}{\mu_{h}}\\ 0\; & -(\mu_{h}+\theta_{h})\; & \frac{\beta_{1}\wedge_{h}}{\mu_{h}}\; & 0\; & 0\; & 0\; & 0\; & \frac{\beta_{2}\wedge_{h}}{\mu_{h}}\\ 0\; & \theta_{h}\; & -(\mu_{h}+\delta +c\tau +(1-c)\tau )\; & 0\; & \rho \; & 0\; & 0\; & 0\\ 0\; & 0\; & c\tau \; & -\mu_{h}\; & 0\; & 0\; & 0\; & 0\\ 0\; & 0\; & (1-c)\tau \; & 0\; & -(\mu_{h}+\rho )\; & 0\; & 0\; & 0\\ 0\; & 0\; & 0\; & 0\; & 0\; & -\mu_{b}\; & 0\; & \frac{-\beta_{3}\wedge_{b}}{\mu_{b}}\\ 0\; & 0\; & 0\; & 0\; & 0\; & 0\; & -(\mu_{b}+\theta_{b})\; & \frac{\beta_{3}\wedge_{b}}{\mu_{b}}\\ 0\; & 0\; & 0\; & 0\; & 0\; & 0\; & \theta_{b}\; & -\mu_{b} \end{array}\right]$$

Let $X=(\mu_{b}+\theta_{b})$, $Y=(\mu_{h}+\delta +c\tau +(1-c)\tau )$, $Z=(\mu_{h}+\rho )$ and $U=(\mu_{b}+\theta_{b})$

(16) $$J=\left[\begin{array}{cccccccc} -\mu_{h}\; & 0\; & \frac{-\beta_{1}\wedge_{h}}{\mu_{h}}\; & 0\; & 0\; & 0\; & 0\; & \frac{-\beta_{2}\wedge_{h}}{\mu_{h}}\\ 0\; & -X\; & \frac{\beta_{1}\wedge_{h}}{\mu_{h}}\; & 0\; & 0\; & 0\; & 0\; & \frac{\beta_{2}\wedge_{h}}{\mu_{h}}\\ 0\; & \theta_{h}\; & -Y\; & 0\; & \rho \; & 0\; & 0\; & 0\\ 0\; & 0\; & c\tau \; & -\mu_{h}\; & 0\; & 0\; & 0\; & 0\\ 0\; & 0\; & (1-c)\tau \; & 0\; & -Z\; & 0\; & 0\; & 0\\ 0\; & 0\; & 0\; & 0\; & 0\; & -\mu_{b}\; & 0\; & \frac{-\beta_{3}\wedge_{b}}{\mu_{b}}\\ 0\; & 0\; & 0\; & 0\; & 0\; & 0\; & -U\; & \frac{\beta_{3}\wedge_{b}}{\mu_{b}}\\ 0\; & 0\; & 0\; & 0\; & 0\; & 0\; & \theta_{b}\; & -\mu_{b} \end{array}\right]$$

(17) $$\left|J-\lambda I\right|=\left[\begin{array}{cccccccc} -\mu_{h}-\lambda \; & 0\; & \frac{-\beta_{1}\wedge_{h}}{\mu_{h}}\; & 0\; & 0\; & 0\; & 0\; & \frac{-\beta_{2}\wedge_{h}}{\mu_{h}}\\ 0\; & -X-\lambda \; & \frac{\beta_{1}\wedge_{h}}{\mu_{h}}\; & 0\; & 0\; & 0\; & 0\; & \frac{\beta_{2}\wedge_{h}}{\mu_{h}}\\ 0\; & \theta_{h}\; & -Y-\lambda \; & 0\; & \rho \; & 0\; & 0\; & 0\\ 0\; & 0\; & c\tau \; & -\mu_{h}-\lambda \; & 0\; & 0\; & 0\; & 0\\ 0\; & 0\; & (1-c)\tau \; & 0\; & -Z-\lambda \; & 0\; & 0\; & 0\\ 0\; & 0\; & 0\; & 0\; & 0\; & -\mu_{b}-\lambda \; & 0\; & \frac{-\beta_{3}\wedge_{b}}{\mu_{b}}\\ 0\; & 0\; & 0\; & 0\; & 0\; & 0\; & -U-\lambda \; & \frac{\beta_{3}\wedge_{b}}{\mu_{b}}\\ 0\; & 0\; & 0\; & 0\; & 0\; & 0\; & \theta_{b}\; & -\mu_{b}-\lambda \end{array}\right]$$

The sole non-negative entry in the first column is $-\mu_{h}-\lambda$, so therefore $\lambda_{1}=-\mu_{h}$.

Step-by-step row operation on subsequent matrices (as exemplified in equations (14)–(17)) gives

$$\lambda_{2}=-\mu_{h},\;\lambda_{3}=-\mu_{b},\;\lambda_{4}=-X=-\left(\mu_{h}+\theta_{h}\right),$$

$$\lambda_{5}=\frac{\theta_{h}\beta_{1}\wedge_{h}}{\mu_{h}}-XY=\frac{\theta_{h}\beta_{1}\wedge_{h}}{\mu_{h}}-(\mu_{h}+\theta_{h})(\mu_{h}+\delta +c\tau +(1-c)\tau ),$$

$$\lambda_{6}=X\rho (1-c)\tau +Z\left(\frac{\theta_{h}\beta_{1}\wedge_{h}}{\mu_{h}}-XY\right)\;\text{which implies}$$

$$\lambda_{6}=\rho \tau (1-c)(\mu_{h}+\theta_{h})+(\mu_{h}+\rho )\left(\frac{\theta_{h}\beta_{1}\wedge_{h}}{\mu_{h}}-(\mu_{h}+\theta_{h})(\mu_{h}+\delta +c\tau +(1-c)\tau )\right)$$

$$\lambda_{7}=-U=-(\mu_{b}+\theta_{b}),\;\lambda_{8}=\frac{\theta_{b}\beta_{3}\wedge_{b}}{\mu_{b}}-\mu_{b}U=\frac{\theta_{b}\beta_{3}\wedge_{b}}{\mu_{b}}-\mu_{b}(\mu_{b}+\theta_{b}).$$

 □

Lemma 4 $R_{G}<1$ *implies the disease-free-equilibrium is locally asymptotically stable.*

Proof We advance from the eigenvalue $\lambda_{6}=\rho \tau (1-c)(\mu_{h}+\theta_{h})+(\mu_{h}+\rho )\left(\frac{\theta_{h}\beta_{1}\wedge_{h}}{\mu_{h}}-(\mu_{h}+\theta_{h})(\mu_{h}+\delta +c\tau +(1-c)\tau )\right)$ and $\lambda_{8}=\frac{\theta_{b}\beta_{3}\wedge_{b}}{\mu_{b}}-\mu_{b}(\mu_{b}+\theta_{b})$ obtained above. We know from equation (11) and (12) that

(18) $$R_{0H}=\frac{\beta_{1}\theta_{h}\Lambda_{h}(\mu_{h}+\rho )}{\left(\mu_{h}+\theta_{h})[(\mu_{h}+\delta +c\tau +(1-c)\tau )(\mu_{h}+\rho )-\rho (1-c)\tau \right]}\;\&\;R_{0B}=\frac{\beta_{3}\theta_{b}\Lambda_{b}}{\mu_{b}^{2}(\mu_{b}+\theta_{b})}$$

$$\lambda_{6}=\rho \tau (1-c)(\mu_{h}+\theta_{h})+(\mu_{h}+\rho )\left(\frac{\theta_{h}\beta_{1}\wedge_{h}}{\mu_{h}}-(\mu_{h}+\theta_{h})(\mu_{h}+\delta +c\tau +(1-c)\tau )\right)=-\left((\mu_{h}+\theta_{h})\{(\mu_{h}+\rho )(\mu_{h}+\delta +c\tau +(1-c)\tau )-\rho \tau (1-c)\}-\frac{\theta_{h}\beta_{1}\wedge_{h}(\mu_{h}+\rho )}{\mu_{h}}\right)=-(\mu_{h}+\theta_{h})\{(\mu_{h}+\rho )(\mu_{h}+\delta +c\tau +(1-c)\tau )-\rho \tau (1-c)\}\left[1-\frac{\beta_{1}\wedge_{h}\theta_{h}(\mu_{h}+\rho )}{\mu_{h}(\mu_{h}+\theta_{h})\{(\mu_{h}+\rho )(\mu_{h}+\delta +c\tau +(1-c)\tau )-\rho \tau (1-c)\}}\right]=-(\mu_{h}+\theta_{h})\{(\mu_{h}+\rho )(\mu_{h}+\delta +c\tau +(1-c)\tau )-\rho \tau (1-c)\}\left[1-R_{0H}\right]\leq -\left[(\mu_{h}+\theta_{h})\{(\mu_{h}+\rho )(\mu_{h}+\delta +c\tau +(1-c)\tau )-\rho \tau (1-c)\}(1-R_{0H})\right]$$

Since $R_{0B}=\frac{\theta_{b}\beta_{3}\wedge_{b}}{\mu_{b}^{2}(\theta_{b}+\mu_{b})}$ and from (18) above:

(19) $$\lambda_{8}=\frac{\theta_{b}\beta_{3}\wedge_{b}}{\mu_{b}}-\mu_{b}(\mu_{b}+\theta_{b})=-(\mu_{b}(\mu_{b}+\theta_{b})-\frac{\theta_{b}\beta_{3}\wedge_{b}}{\mu_{b}})=-\left(\mu_{b}(\mu_{b}+\theta_{b})\right)\left[1-\frac{\theta_{b}\beta_{3}\wedge_{b}}{\mu_{b}^{2}(\mu_{b}+\theta_{b})}\right]=-(\mu_{b}(\mu_{b}+\theta_{b}))\left[1-R_{0B}\right]\leq -\mu_{b}(\mu_{b}+\theta_{b})(1-R_{0B})$$

From (19) above, Eigenvalues $\lambda_{6}$ and $\lambda_{8}$ remain negative justifying that $R_{0B}<1$ and $R_{0H}<1$ in Theorem 3. And since $R_{0B}<1$ and $R_{0H}<1$, then $R_{{}_{G}}^{2}=R_{0B}\times R_{0H}<1$. Therefore $R_{G}<1$. □

### 5.2 Model global stability analysis

Theorem 5 $R_{G}<1$ *implies the disease-free-equilibrium (DFE) of system* (2) *–* (3) *is globally stable on M.*

Proof To prove this important theorem, we implement the method used in [29], [30].

We start by dividing the system of equation (2)–(3) into two parts and also define some new variables.

Let

(20) $$X=\left(S_{h},R_{his},R_{hni},S_{b}\right)\;\text{and}\;I=\left(E_{h},I_{h},E_{b},I_{b}\right)$$

(21) $$\frac{dX}{dt}=F(X,I)\;\text{and}\;\frac{dI}{dt}=G(X,I),\;G(X,0)=0$$

Where the number of infection free compartment is denoted by $X\in {ℜ}^{4}$ and $I\in {ℜ}^{4}$ is the numbers of infection compartments. The double vector value functions F(X,I) and G(X,I) are defined as:

(22) $$F(X,I)=\left[\begin{array}{c} \wedge_{h}-\mu_{h}S_{h}-\phi_{1}S_{h}\\ c\tau I_{h}-\mu_{h}R_{his}\\ (1-c)\tau I_{h}-(\mu_{h}+\rho )R_{hni} \end{array}\right]$$

(23) $$G(X,I)=\left[\begin{array}{c} \phi_{1}S_{h}-(\mu_{h}+\theta_{h})E_{h}\\ \theta_{h}E_{h}+\rho R_{hni}-(\mu_{h}+\delta +c\tau +(1-c)\tau )I_{h}\\ \phi_{2}S_{b}-(\mu_{b}+\theta_{b})E_{b}\\ \theta_{b}E_{b}-\mu_{b}I_{b} \end{array}\right]$$

With G(X,0)=0 and $E^{0}=(X^{⁎},0)$ representing the disease-free equilibrium of the subsystems $X^{⁎}=\left(\frac{\wedge_{h}}{\mu_{h}},0.0,\frac{\wedge_{b}}{\mu_{b}}\right)$. The conditions $H_{1}$ and $H_{2}$ below need be totally satisfied for global stability to hold.

(24) $$H_{1}:\left(\frac{dX}{dt}\right)=F(X,0)\;\text{and}\;H_{2}:G(X,I)=BI-\overset{ˆ}{G}(X,I)$$

With *B* as an *M*-matrix where the off-diagonal element is not negative.

When we consider the system that were reduced, $\left(\frac{dX}{dt}\right)=F(X,0)$, i.e. I=0. We have that:

(25) $$\begin{array}{c} \frac{dS_{h}}{dt}=\wedge_{h}-\mu_{h}S_{h}\\ \frac{dR_{hni}}{dt}=-\mu_{h}R_{his}\\ \frac{dR_{his}}{dt}=-(\mu_{h}+\rho )R_{hni}\\ \frac{dS_{b}}{dt}=\wedge_{b}-\mu_{b}S_{b} \end{array}\;]$$

$X^{⁎}=\left(S_{h},R_{his},R_{his},S_{b}\right)=\left(\frac{\wedge_{h}}{\mu_{h}},0,0,\frac{\wedge_{b}}{\mu_{b}}\right)$ is the GAS equilibrium point for our reduced system $\frac{dX}{dt}=F(X,0)$ of (21).

The solution of $X^{⁎}$ has earlier been obtained while finding the positive invariant region. At t→∞, we obtain the respective values at DFE. Hence, we conclude the convergence of solutions of equations (29)–(31) is global in *M* and so:

(26) $$G(X,I)=\left[BI-\overset{ˆ}{G}(X,I\right)\text{ with }\overset{ˆ}{G}(X,I)\geq 0,\;\forall (X,I)\in M]$$

Where

(27) $$B=\left[\begin{array}{cccc} -(\mu_{h}+\theta_{h})\; & \frac{\beta_{1}\wedge_{h}}{\mu_{h}}\; & 0\; & \frac{\beta_{2}\wedge_{b}}{\mu_{b}}\\ \theta_{h}\; & -(\mu_{h}+\delta +c\tau +(1-c)\tau )\; & 0\; & 0\\ 0\; & 0\; & -(\mu_{b}+\theta_{b})\; & \frac{\beta_{3}\wedge_{b}}{\mu_{b}}\\ 0\; & 0\; & \theta_{b}\; & -\mu_{b} \end{array}\right]$$

$$I=\left[\begin{array}{c} E_{h}\\ I_{h}\\ E_{b}\\ I_{b} \end{array}\right],\;\overset{ˆ}{G}(X,I)=\left[\begin{array}{c} \left(\frac{\beta_{1}\wedge_{h}}{\mu_{h}}+\frac{\beta_{2}\wedge_{b}}{\mu_{b}})(\frac{\wedge_{h}}{\mu_{h}}-S_{h}\right)\\ \frac{\beta_{3}\wedge_{b}}{\mu_{b}}\left(\frac{\wedge_{b}}{\mu_{b}}-S_{b}\right) \end{array}\right]$$

Equations (20)–(27) took us through the established process of proving the condition for global stability of the model. □

#### 5.2.1 The Endemic Equilibrium State (EES)

Theorem 6 *System* (2) *–* (3) *express a distinct EES,* $E^{⁎}=\{S_{h}^{⁎},E_{h}^{⁎},I_{h}^{⁎},R_{hni}^{⁎},R_{his}^{⁎},S_{b}^{⁎},E_{b}^{⁎},I_{b}^{⁎}\}$ *if* $R_{G}>1$ *.*

At equilibrium; $\frac{dS_{h}}{dt}=\frac{dE_{h}}{dt}=\frac{dI_{h}}{dt}=\frac{dR_{hni}}{dt}=\frac{dR_{his}}{dt}=\frac{dS_{b}}{dt}=\frac{dE_{b}}{dt}=\frac{dI_{b}}{dt}=0$.

From the system of equations (2)–(3).

Let $\frac{dS_{b}}{dt}=\frac{dE_{b}}{dt}=\frac{dI_{b}}{dt}=0$, then we have:

(28) $$\wedge_{b}-(\mu_{b}+\phi_{2})S_{b}=0$$

(29) $$\phi_{2}S_{b}-(\mu_{b}+\theta_{b})E_{b}=0$$

(30) $$\theta_{b}E_{b}-\mu_{b}I_{b}=0$$

Recall $\phi_{2}=\frac{\beta_{3}I_{b}}{1+mI_{b}}$ at m=0 for feasibility, and then we have:

From (30), we get

(31) $$E_{b}=\frac{\mu_{b}I_{b}}{\theta_{b}}$$

Putting (29) into (30) gives

$$I_{b}\left[\beta_{3}S_{b}-\frac{(\mu_{b}+\theta_{b})\mu_{b}}{\theta_{b}}\right]=0\;\Rightarrow \;I_{b}=0\text{ or }\beta_{3}S_{b}-\frac{(\mu_{b}+\theta_{b})\mu_{b}}{\theta_{b}}=0$$

Therefore,

(32) $$S_{b}=\frac{(\mu_{b}+\theta_{b})\mu_{b}}{\beta_{3}\theta_{b}}$$

Systematic substitution where necessary (in (28), (32) and so on) give solution (34) below:

(33) $$E^{⁎}(S_{h}^{⁎},E_{h}^{⁎},R_{hni}^{⁎},R_{his}^{⁎},S_{b}^{⁎},E_{b}^{⁎},)=\left(\begin{array}{c} (\mu_{h}+\theta_{h})\left[\frac{\left(\mu_{h}+\delta +c\tau +(1-c)\tau )(\mu_{h}+\rho )-\rho \tau (1-c\right)}{\beta_{1}(\mu_{h}+\rho )}\right]\\ \frac{\left[(\mu_{h}+\delta +c\tau +(1-c)\tau )(\mu_{h}+\rho )-\rho \tau (1-c)\right]I_{h}^{⁎}}{\left(\mu_{h}+\rho \right)}\\ \frac{(1-c)\tau I_{h}^{⁎}}{\left(\mu_{h}+\rho \right)}\\ \frac{c\tau I_{h}^{⁎}}{\mu_{h}}\\ \frac{(\mu_{b}+\theta_{b})\mu_{b}}{\beta_{3}\theta_{b}}\\ \frac{\mu_{b}I_{b}^{⁎}}{\theta_{b}} \end{array}\right)$$

Working through equations (28)–(33), we have proven the condition necessary for existence of EES.

## 6 Optimal control analysis

Recall from (1) that $\phi_{1}=\frac{\beta_{1}I_{h}}{1+mI_{h}}+\frac{\beta_{2}I_{b}}{1+mI_{b}}$ and $\phi_{2}=\frac{\beta_{3}I_{b}}{1+mI_{b}}$.

We now introduce control measures $k_{1},k_{2},k_{3}$, where:

$k_{1}=$total abstinent from touching a patient who died of Ebola virus disease irrespective of closeness or family or social ties/rites to the deceased.

$k_{2}=$deliberate depopulation of bats in the urban areas by a specialized team and an outright ban on hunting bats for food to curtail transmission.

$k_{3}=$Immediate treatment of the infected in isolation.

Let *σ* the probability of infection, the system (2) for human becomes:

(34) $$\begin{array}{c} \frac{dS_{h}}{dt}=\wedge_{h}-\mu_{h}S_{h}-\frac{(1-k_{1})\sigma \beta_{1}I_{h}S_{h}}{1+mI_{h}}-\frac{(1-k_{2})\sigma \beta_{2}I_{b}S_{h}}{1+mI_{b}}\\ \frac{dE_{h}}{dt}=\frac{(1-k_{1})\sigma \beta_{1}I_{h}S_{h}}{1+mI_{h}}+\frac{(1-k_{2})\sigma \beta_{2}I_{b}S_{h}}{1+mI_{b}}+(\mu_{h}+\theta_{h})E_{h}\\ \frac{dI_{h}}{dt}=\theta_{h}E_{h}+\rho R_{hni}-(\mu_{h}+\delta +c\tau +(1-c)\tau )I_{h}-k_{3}I_{h}\\ \frac{dR_{his}}{dt}=k_{3}I_{h}+c\tau I_{h}-\mu_{h}R_{his}\\ \frac{dR_{hni}}{dt}=k_{3}I_{h}+(1-c)\tau I_{h}-(\mu_{h}+\rho )R_{hni} \end{array}\;]$$

### 6.1 Mathematical analysis of the model with control measures

Using Pontryagin's maximum principle [31], [36], an objective functional is formulated and we present the existence of the optimal control. The objective functional (*H*) establishes the most effective of the strategies presented: complete abstaining from touching the deceased from Ebola infection (burial should be handled by established professional agencies), an outright ban on bat hunting for food, and deliberate depopulation of bats in cities by specialized teams and immediate treatment of the infected individual in isolation. These measures are analytically presented to be feasible in minimizing the transmission of this deadly virus in a finite time interval [0,T] with $K=\{(k_{1},k_{2},k_{3})\in K\}$ Lebesgue measurable on [0,1], $0\leq k_{i}(t)\leq 1\in [0,T]$, i=1,2,3.

We define the objective functional *H* as follows:

(35) $$H(k_{1},k_{2},k_{3})=\int_{0}^{T}(M_{1}I+M_{2}E+\frac{1}{2}(W_{1}k_{1}^{2}+W_{2}k_{2}^{2}+W_{3}k_{3}^{2}))dt$$

Which is subject to the system (34) with $S_{h}(0)≻0$, $E_{h}(0)≻0$, $I_{h}(0)≻0$, $R_{his}(0)≻0$, $R_{hni}(0)≻0$, $S_{b}(0)≻0$, $E_{b}(0)≻0$ and $I_{b}(0)≻0$.

Where $k_{1}$ is the total abstinent of loved ones from the remains of Ebola victims, $k_{2}$, is the deliberate depopulation or remigration of bats from the cities by a specialized team to avoid hunting them for food by virtue of their proximity, and $k_{3}$ is the immediate isolation of EVD patients for treatment.

In the objective function, *T* is the final time and parameters $M_{1}$, $M_{2}$ are weight constants of the virus in the environment, infected class, exposed class, and the hospitalized respectively.

To achieve the objective of the control problem, we implement the function such that

(36) $$H(k_{1}^{⁎}(t),k_{2}^{⁎}(t),k_{3}^{⁎}(t))=\min\{H(k_{1},k_{2},k_{3}),k_{1},k_{2},k_{3}\in K\}$$

### 6.2 Existence of an optimal control

Theorem 7 *Considering the objective functional* $H(k_{1},k_{2},k_{3})$ *as in* (36) *above where the control set K is measurable subject to* (34) *with the initial condition given at* t=0 *, then there exists an optimal control* $K^{⁎}=(k_{1}^{⁎}(t),k_{2}^{⁎}(t),k_{3}^{⁎}(t))$ *such that* $H(k_{1}^{⁎}(t),k_{2}^{⁎}(t),k_{3}^{⁎}(t))=\min\{H(k_{1},k_{2},k_{3}),k_{1},k_{2},k_{3}\in K\}$ *.*

Proof Resulting from the convexity of the integral of *H* to optimize control $k_{1}$, $k_{2}$, $k_{3}$, the non-negative invariant region of the model, boundedness of solution, and the Lipchitz property of the system of the model concerning the state variables $S_{h}$, $E_{h}$, $I_{h}$, $R_{his}$, $R_{hni}$, $S_{b}$, $E_{b}$, $I_{b}$, then the optimal control of the model exist.

Now we will need to establish the Lagrangian (*L*) and Hamiltonian (*H*) for the optimal control problem.

We write the Lagrangian as

(37) $$L=M_{1}I+M_{2}E+\frac{1}{2}(W_{1}k_{1}^{2}+W_{2}k_{2}^{2}+W_{3}k_{3}^{2})$$

And the Hamiltonian function for the system is:

(38) $$H=M_{1}I_{h}+M_{2}E_{h}+\frac{1}{2}(W_{1}k_{1}^{2}+W_{2}k_{2}^{2}+W_{3}k_{3}^{2})+\alpha_{TS_{h}}\left[\wedge_{h}-\mu_{h}S_{h}-\frac{(1-k_{1})\sigma \beta_{1}I_{h}S_{h}}{1+mI_{h}}-\frac{(1-k_{2})\sigma \beta_{2}I_{b}S_{h}}{1+mI_{b}}\right]+\alpha_{TE_{h}}\left[\frac{(1-k_{1})\sigma \beta_{1}I_{h}S_{h}}{1+mI_{h}}+\frac{(1-k_{2})\sigma \beta_{2}I_{b}S_{h}}{1+mI_{b}}-(\mu_{h}+\theta_{h})E_{h}\right]+\alpha_{TI_{h}}\left[\theta_{h}E_{h}+\rho R_{hni}-(\mu_{h}+\delta +c\tau +(1-c)\tau )I_{h}-k_{3}I_{h}\right]+\alpha_{TR_{his}}\left[k_{3}I_{h}+c\tau I_{h}-\mu_{h}R_{his}\right]+\alpha_{TR_{hni}}\left[k_{3}I_{h}+(1-c)\tau I_{h}-(\mu_{h}+\rho )R_{hni}\right]+\alpha_{TS_{b}}\left[\wedge_{b}-(\mu_{b}+\phi_{2})S_{b}\right]+\alpha_{TE_{b}}\left[\phi_{2}S_{b}-(\mu_{b}+\theta_{b})E_{b}\right]+\alpha_{TI_{b}}\left[\theta_{b}E_{b}-\mu_{b}I_{b}\right]$$

Where $\alpha_{T_{i}}$, $i\in \left\{S_{h},E_{h},I_{h},R_{his},R_{hni},S_{b,}E_{b},I_{b}\right\}$ are the disjoint variables and $\phi_{2}=\frac{\beta_{3}I_{b}}{1+mI_{b}}$.

We now apply the necessary conditions in Theorem 7 to the Hamiltonian – *H*. □

Theorem 8 *Considering an optimal control* $K^{⁎}=(k_{1}^{⁎}(t),k_{2}^{⁎}(t),k_{3}^{⁎}(t))$ *and a solution* $Z^{⁎}=\{S_{h}^{⁎},E_{h}^{⁎},I_{h}^{⁎},R_{his}^{⁎},R_{hni}^{⁎}S_{b}^{⁎},E_{b}^{⁎},I_{b}^{⁎}\}$ *of the existing model of the system* (2) *–* (3) *, then there exists an adjoint variable* $\alpha_{T_{i}}$ *where* $i\in \left\{S_{h},E_{h},I_{h},R_{his},R_{hni},S_{b,}E_{b},I_{b}\right\}$ *satisfying*

(39) $$\frac{d\alpha_{TS_{h}}}{dt}=\left[\alpha_{TS_{h}}\left[\frac{(1-k_{1})\sigma \beta_{1}I_{h}}{1+mI_{h}}+\frac{(1-k_{2})\sigma \beta_{2}I_{b}}{1+mI_{b}}-\mu_{h}\right]-\alpha_{TE_{h}}\left[\frac{(1-k_{1})\sigma \beta_{1}I_{h}}{1+mI_{h}}+\frac{(1-k_{2})\sigma \beta_{2}I_{b}}{1+mI_{b}}\right]\right]$$

$$\frac{d\alpha_{TE_{h}}}{dt}=-M_{2}+\left[\alpha_{TE_{h}}(\mu_{h}+\theta_{h})-\alpha_{TI_{h}}\theta_{h}\right]$$

$$\frac{d\alpha_{TI_{h}}}{dt}=-M_{1}+\left[\begin{array}{c} \alpha_{TI_{h}}((\mu_{h}+\delta +c\tau +(1-c)\tau +k_{3})-\alpha_{TR_{his}}(k_{3}+c\tau )-\alpha_{TR_{hni}}(k_{3}+(1-c)\tau )\\ +\alpha_{TS_{h}}\left[\frac{\sigma \beta_{1}S_{h}(1-k_{1})}{{\left(1+mI_{h}\right)}^{2}}\right]-\alpha_{TE_{h}}\left[\frac{\sigma \beta_{1}S_{h}(1-k_{1})}{{\left(1+mI_{h}\right)}^{2}}\right] \end{array}\right]$$

$$\frac{d\alpha_{TR_{his}}}{dt}=\alpha_{TR_{his}}(\mu_{h})$$

$$\frac{d\alpha_{TR_{hni}}}{dt}=\left[-\alpha_{TI_{h}}(\rho )+\alpha_{TR_{hni}}(\mu_{h}+\rho )\right]$$

$$\frac{d\alpha_{TS_{b}}}{dt}=\left[\alpha_{TS_{b}}\left(\frac{\beta_{3}I_{b}}{1+mI_{b}}+\mu_{b}\right)-\alpha_{TE_{b}}\left(\frac{\beta_{3}I_{b}}{1+mI_{b}}\right)\right]$$

$$\frac{d\alpha_{TE_{b}}}{dt}=\left[\alpha_{TE_{b}}\left(\mu_{b}+\theta_{b}\right)-\alpha_{TI_{b}}\theta_{b}\right]$$

$$\begin{array}{cc} \frac{d\alpha_{TI_{b}}}{dt}= & \alpha_{TS_{h}}\left[\frac{\sigma \beta_{2}S_{h}(1-k_{2})}{{\left(1+mI_{b}\right)}^{2}}\right]-\alpha_{TE_{h}}\left[\frac{\sigma \beta_{2}S_{h}(1-k_{2})}{{\left(1+mI_{b}\right)}^{2}}\right]+\alpha_{TS_{b}}\left[\frac{\beta_{3}}{{\left(1+mI_{b}\right)}^{2}}\right]\\ & -\alpha_{TE_{b}}\left[\frac{\beta_{3}}{{\left(1+mI_{b}\right)}^{2}}\right]+\alpha_{TI_{b}}(\mu_{b}) \end{array}$$

*With transversality conditions* $\alpha_{T_{i}}$ *,* $i(T)\in \left\{S_{h},E_{h},I_{h},R_{his},R_{hni},S_{b,}E_{b},I_{b}\right\}$ *which imply the control functions are:*

$$k_{1}^{⁎}=\min\left\{1,\max\left\{0,\gamma_{1}\right\}\right\},\;k_{2}^{⁎}=\min\left\{1,\max\left\{0,\gamma_{2}\right\}\right\}\;k_{3}^{⁎}=\min\left\{1,\max\left\{0,\gamma_{3}\right\}\right\}$$

*Where*

(40) $$\gamma_{1}=\frac{(\alpha_{TE_{h}}-\alpha_{TS_{h}})\sigma \beta_{1}S_{h}I_{h}}{W_{1}(1+mI_{h})}+\frac{(\alpha_{TE_{h}}-\alpha_{TS_{h}})\sigma \beta_{1}S_{h}I_{h}}{W_{1}{\left(1+mI_{h}\right)}^{2}}$$

$$\gamma_{2}=\frac{(\alpha_{TE_{h}}-\alpha_{TS_{h}})\sigma \beta_{2}S_{h}I_{b}}{W_{2}(1+mI_{b})}+\frac{(\alpha_{TE_{h}}-\alpha_{TS_{h}})\sigma \beta_{2}S_{h}I_{b}}{W_{2}{\left(1+mI_{b}\right)}^{2}}$$

$$\gamma_{3}=\frac{(\alpha_{TI_{h}}-\alpha_{TR_{his}}-\alpha_{TR_{hni}})I_{h}}{W_{3}}$$

*To obtain the adjoint equation and the conditions for transversality, we differentiate the Hamiltonian with respect to the state variables and obtain the following equation:*

(41) $$\frac{d\alpha_{TS_{h}}}{dt}=-\frac{\partial H}{dS_{h}}=\left[\alpha_{TS_{h}}\left(\frac{(1-k_{1})\sigma \beta_{1}I_{h}}{1+mI_{h}}+\frac{(1-k_{2})\sigma \beta_{2}I_{b}}{1+mI_{b}}-\mu_{h}\right)-\alpha_{TE_{h}}\left(\frac{(1-k_{1})\sigma \beta_{1}I_{h}}{1+mI_{h}}+\frac{(1-k_{2})\sigma \beta_{2}I_{b}}{1+mI_{b}}\right)\right]$$

$$\frac{d\alpha_{TE_{h}}}{dt}=-\frac{\partial H}{dE_{h}}=-M_{2}+\left[\alpha_{TE_{h}}(\mu_{h}+\theta_{h})-\alpha_{TI_{h}}\theta_{h}\right]$$

$$\frac{d\alpha_{TI_{h}}}{dt}=-\frac{\partial H}{dI_{h}}=-M_{1}+\left[\begin{array}{c} \alpha_{TI_{h}}\left((\mu_{h}+\delta +c\tau +(1-c)\tau )+k_{3}\right)-\alpha_{TR_{his}}(k_{3}+c\tau )\\ -\alpha_{TR_{hni}}(k_{3}+(1-c)\tau )+\alpha_{TS_{h}}\left(\frac{\sigma \beta_{1}S_{h}(1-k_{1})}{{\left(1+mI_{h}\right)}^{2}}\right)-\alpha_{TE_{h}}\left(\frac{\sigma \beta_{1}S_{h}(1-k_{1})}{{\left(1+mI_{h}\right)}^{2}}\right) \end{array}\right]$$

$$\frac{d\alpha_{TR_{his}}}{dt}=-\frac{\partial H}{dR_{his}}=\alpha_{TR_{his}}\mu_{h}$$

$$\frac{d\alpha_{TR_{hni}}}{dt}=-\frac{\partial H}{dR_{hni}}=-\alpha_{TI_{h}}\rho +\alpha_{TR_{hni}}(\mu_{h}+\rho )$$

$$\frac{d\alpha_{TS_{b}}}{dt}=-\frac{\partial H}{dS_{b}}=\alpha_{TS_{b}}\left(\frac{\beta_{3}I_{b}}{1+mI_{b}}+\mu_{b}\right)-\alpha_{T}E_{b}\left(\frac{\beta_{3}I_{b}}{1+mI_{b}}\right)$$

$$\frac{d\alpha_{TE_{b}}}{dt}=-\frac{\partial H}{dE_{b}}=\alpha_{TE_{b}}(\mu_{b}+\theta_{b})-\alpha_{TI_{b}}\theta_{b}$$

$$\frac{d\alpha_{TI_{b}}}{dt}=-\frac{\partial H}{dI_{b}}=\left[\begin{array}{c} \alpha_{TS_{h}}\left(\frac{\sigma \beta_{2}S_{h}(1-k_{2})}{{\left(1+mI_{b}\right)}^{2}}\right)\\ -\alpha_{TE_{h}}\left(\frac{\sigma \beta_{2}S_{h}(1-k_{2})}{{\left(1+mI_{b}\right)}^{2}}\right)+\alpha_{TS_{b}}\left(\frac{\beta_{3}}{{\left(1+mI_{b}\right)}^{2}}\right)\\ -\alpha_{TE_{b}}\left(\frac{\beta_{3}}{{\left(1+mI_{b}\right)}^{2}}\right)+\alpha_{TI_{b}}\mu_{b} \end{array}\right]$$

*With the conditions for transversality* $\alpha_{T_{i},}i(T)\in \{S_{h},E_{h},I_{h},R_{his},R_{hni},S_{b},E_{b},I_{b}\}$ *. To reduce the Hamiltonian (H) with regards to the optimal controls, we apply the idea of differentiation, i.e., H is differentiated with respect to* $k_{1}$ *,* $k_{2}$ *and* $k_{3}$ *.*

Equating the results to zero gives the solution. This provides the required optimization measures and putting in the boundary condition of each of this control gives:

$$k_{1}^{⁎}=\min\left\{1,\max\left\{0,\gamma_{1}\right\}\right\},\;k_{2}^{⁎}=\min\left\{1,\max\left\{0,\gamma_{2}\right\}\right\}\;k_{3}^{⁎}=\min\left\{1,\max\left\{0,\gamma_{3}\right\}\right\}$$

Where

$$\gamma_{1}=\frac{(\alpha_{TE_{h}}-\alpha_{TS_{h}})\sigma \beta_{1}S_{h}I_{h}}{W_{1}(1+mI_{h})}+\frac{(\alpha_{TE_{h}}-\alpha_{TS_{h}})\sigma \beta_{1}S_{h}I_{h}}{W_{1}{\left(1+mI_{h}\right)}^{2}}$$

$$\gamma_{2}=\frac{(\alpha_{TE_{h}}-\alpha_{TS_{h}})\sigma \beta_{2}S_{h}I_{b}}{W_{2}(1+mI_{b})}+\frac{(\alpha_{TE_{h}}-\alpha_{TS_{h}})\sigma \beta_{2}S_{h}I_{b}}{W_{2}{\left(1+mI_{b}\right)}^{2}}$$

$$\gamma_{3}=\frac{(\alpha_{TI_{h}}-\alpha_{TR_{his}}-\alpha_{TR_{hni}})I_{h}}{W_{3}}$$

From equation (34)–(40), we have been able to prove the necessary condition for the existence of optimal control, and so this completes the proof. Hence our model (2) and (3) progressively satisfied all the conditions listed above. These justify the feasibilities of the new proposed model on mathematical modeling of Ebola virus.

## 7 Discussion and interpretation

In this study, we created a mathematical model for the dynamic spread of the Ebola virus disease, and we included three preventative strategies. We examined and presented the mathematical analysis of the model with (out) the control measures. The stability analysis carried out showed that the disease-free equilibrium is locally asymptotically stable if $R_{G}<1$. And we stated the endemic equilibrium which is globally asymptotically stable if $R_{G}>1$. Using MATLAB – ODE 45 software, we have succinctly shown the effect of the control strategies on each compartment. Table 1 and Table 2 presented the state ad parameter values used for the simulation.

**Table 1:** 

State variables	Values	Sources
*S*_*h*_	6000	Estimated
*E*_*h*_	4300	Assumed
*I*_*h*_	990	Assumed
*R*_*his*_	500	Estimated
*R*_*hni*_	150	Estimated
*S*_*b*_	100	Assumed
*E*_*b*_	80	Assumed
*I*_*b*_	35	Assumed

**Table 2:** 

Parameters	Value	Dimension	Source
Λ_*h*_	1.2	Humans *x* day^−1^	Roughly Calculated
Λ_*b*_	0.7	Bat *x* day^−1^	Roughly Calculated
*β*_1_	0.04–0.004	No dimension	Presumed
*β*_2_	0.04–0.004	No dimension	Assumed
*β*_3_	0.005–0.000005	No dimension	Presumed
*θ*_*h*_	0.1–0.0001	Day^−1^	[26]
*θ*_*b*_	0.057	Day^−1^	[26]
*τ*	0.03	Inmazeb *x* day^−1^	[15]
		Ebanga *x* day^−1^	
*ρ*	0.0085	Day^−1^	[27]
*δ*	0.0185	Day^−1^	Presumed
*μ*_*h*_	0.0003986	Day^−1^	Roughly Calculated
*μ*_*b*_	0.00261026	Day^−1^	[29], [30]
*ϕ*_1_	0.091193	No dimension	Roughly Calculated
*ϕ*_2_	0.32	No dimension	Roughly Calculated
*m*	0.83	No dimension	Presumed
*c*	0.1–0.9	No dimension	Presumed

The following is a summary of the potential impacts of the included control strategies.

**Figure 3:**
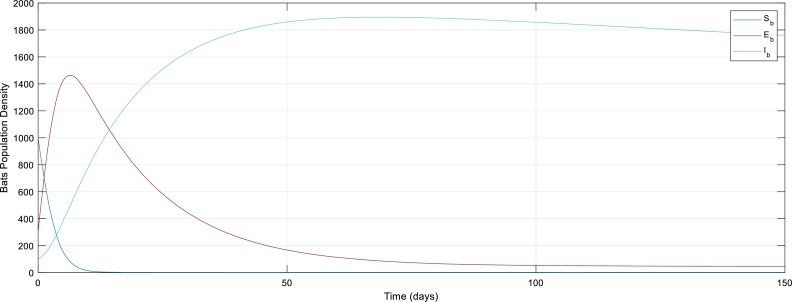
The graph of Bat population without control strategies.

Fig. 3: The mathematical modeling dynamics of the $S_{h}E_{h}I_{h}R_{his}R_{hni}-S_{b}E_{b}I_{b}$ model considering only the Bat population before the intervention of control strategies. From the graph, we observed the population of bats grows where there are no control strategies.

**Figure 4:**
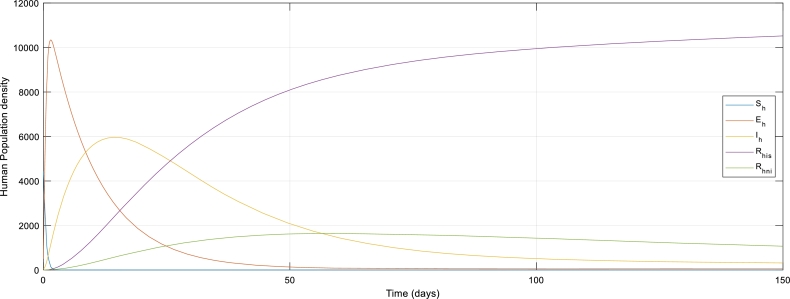
The graph of Human population without control strategies.

Fig. 4: The mathematical modeling dynamics of the $S_{h}E_{h}I_{h}R_{his}R_{hni}-S_{b}E_{b}I_{b}$ model considering only the Human population before the intervention of control strategies. The graph revealed that the susceptible population steadily reduced as people get exposed to the virus.

**Figure 5:**
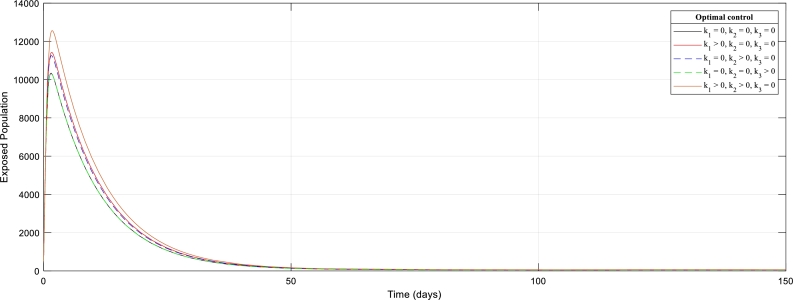
The graph of Exposed Human population with (out) control strategies.

Fig. 5: shows the effect of the control strategies in the Exposed population. It is observed that the most effective control measure in the Exposed population is the immediate treatment of the Ebola patient whenever there is an outbreak. There is an absolute superimposition of the immediate treatment curve with the curve without control measures. Other effects are clearly shown on the graph

**Figure 6:**
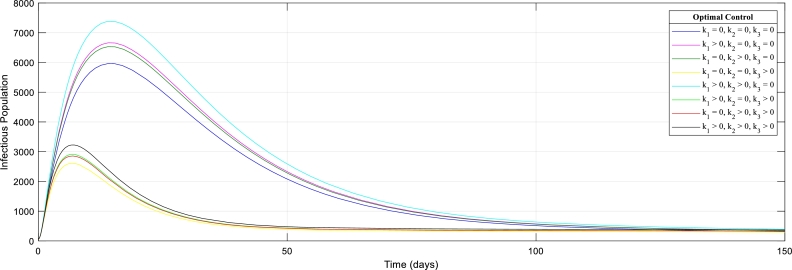
The graph of Infectious Human population with (out) control strategies.

Fig. 6: shows the effect of control strategies in the Infectious population. Results clearly showed the effectiveness of the possible combination of the control strategies in stemming the transmission of this deadly virus. The curves showed that the immediate treatment of the infectious individuals is the most effective control strategy and the remaining control strategies follow immediately.

**Figure 7:**
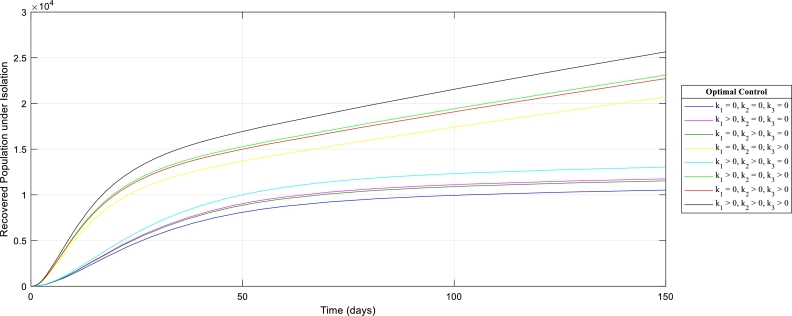
The graph of the Recovered Human population in isolation with (out) control strategies.

Fig. 7: shows the effect of control strategies ($k_{1},k_{2},k_{3}$) in the Recovered population in isolation. It is observed that implementing the three control measures simultaneously would drastically increase this population and subsequently present a healthy population.

**Figure 8:**
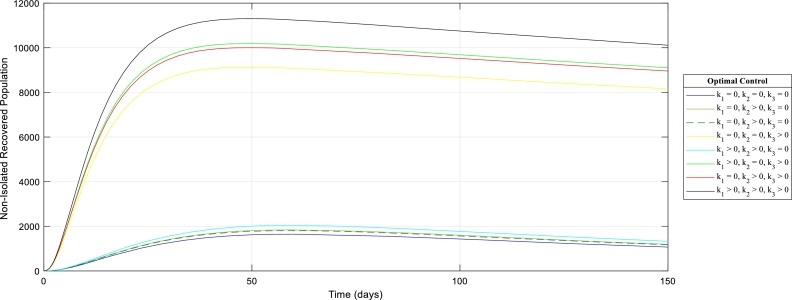
The graph of the Recovered Human population in non-isolation with (out) control strategies.

Fig. 8: reveals that in the presence of the three control strategies ($k_{1},k_{2},k_{3}$), the human in non-isolation will totally recover after some time. Also, combining one or two of the measures will gradually lead to the recovery of all the humans in non-isolation. Hence, the population may attain a disease-free equilibrium through the implementation of the aforementioned control measures.

## 8 Conclusion

Earlier research concentrated on within-host transmission dynamics and possible control strategies for the Ebola virus. In this study, we presented a novel vector-host mathematical model, particularly the human-bat model which forecasts the transmission and severity of the Ebola virus from bats to human to analytical study the impact of some control measures that would help stamp out the transmission of Ebola virus. We considered strategies which include systematic and deliberate depopulation of bats (through persecution, pet capturing, and roosts destruction) from the cities to avoid hunting them for food by virtue of their proximity, immediate isolation of infected individuals for treatment in case of an outbreak and complete abstaining from touching the remains of patients who died from Ebola virus irrespective of their social affiliation hence allowing designated agencies to handle the burial to prevent post-death transmission. We showed the presence of optimal control, proved the feasibility of the model by validating parameters cogent in mathematical epidemiology, and also presented results that remarkably show that the front liners and government agencies are better informed in their decision making as to the possible control strategies to adopted in their battle against Ebola virus in a vector – host situation to arrive at a healthy population.

## Declarations

### Author contribution statement

Joshua Oluwasegun Agbomola: Conceived and designed the analysis.

Adedapo Chris Loyinmi: Analyzed and interpreted the data; Contributed analysis tools or data; Wrote the paper.

### Funding statement

This research did not receive any specific grant from funding agencies in the public, commercial, or not-for-profit sectors.

### Data availability statement

Data will be made available on request.

### Declaration of interests statement

The authors declare no conflict of interest.

### Additional information

No additional information is available for this paper.
